# Temperature contributes to host specialization of coffee wilt disease (*Fusarium xylarioides*) on arabica and robusta coffee crops

**DOI:** 10.1038/s41598-023-36474-w

**Published:** 2023-06-08

**Authors:** Xiuhan Zhang, Lily D. Peck, Julie Flood, Matthew J. Ryan, Timothy G. Barraclough

**Affiliations:** 1 Department of Life Sciences, Imperial College London, Silwood Park Campus, Ascot, Berkshire, SL5 7PY UK; 2 CABI, Bakeham Lane, Egham, TW20 9TY Surrey UK; 3 Department of Biology, University of Oxford, 11a Mansfield Rd, Oxford, OX1 3SZ UK

**Keywords:** Agroecology, Climate-change ecology, Fungal biology

## Abstract

Coffee wilt disease, caused by the fungus *Fusarium xylarioides*, is a vascular wilt disease that has affected coffee production in sub-Saharan Africa over the past century. Today, the disease has two host-specific populations specialising on arabica and robusta coffee crops, which grow at high and low altitude, respectively. Here we test whether adaptation to different temperatures contributes to specialisation of the fungi on each crop. Firstly, climate models show that the severity of the arabica and robusta populations of coffee wilt disease correlates with temperature. The robusta population shows higher peak severity than the arabica population overall, but the latter has greater cold tolerance. Secondly, growth assays of thermal performance of fungal strains in vitro show that, while robusta strains grow faster than arabicas at intermediate temperatures, the arabica strains have higher sporulation and spore germination rates at temperatures below 15ºC. The match between environmental patterns of severity in nature with thermal performance of fungal cultures in the laboratory supports a role for temperature adaptation in specialisation on arabica and robusta coffee. Extrapolating our temperature-models to future climate change predicts that disease severity could decline on average due to increased temperature but could increase in some coffee-growing regions.

## Introduction

Ongoing climate change is an increasing threat to agricultural yields in many countries, including major crops such as wheat and coffee^1,2^. Changes in key climatic variables such as temperature not only affect growth and production of crop plants directly^3^ but also, indirectly, by influencing the infectivity and pathogenicity of crop diseases^4^. Predicting the impacts of global warming on agricultural production and planning adaptation strategies for future scenarios therefore requires better understanding of the effects of temperature on different crops and crop diseases^4^.

Coffee is one of the most valuable agricultural commodities in the world^5^. Its retail value was estimated to be $108 billion in 2021, accounting for around 0.5% of world merchandise trade each year^6,7^. Today, most coffee is produced from two *Coffea* species: *C. arabica*, known as arabica coffee, and *C. canephora* robusta, known as robusta coffee, which account for approximately 60% and 40% of world coffee production, respectively^8^. Arabica coffee is more susceptible to diseases and climatic variations but also considered better quality than robusta coffee, which is mostly used in instant coffee^9^. Globally, most coffee production takes place in developing countries, especially in South America, Southeast Asia, and Africa, supporting the livelihoods of hundreds of millions of people^10^. The coffee industry is vital to some of these countries, including Ethiopia, where coffee export can account for over 80% of foreign currency income, and therefore plays an important role in world economy and development^10,11^.

Coffee wilt disease (CWD), also known as tracheomycosis, is a vascular wilt disease affecting several *Coffea* species. The disease is caused by the fungus *Fusarium xylarioides* Steyaert (anamorph: *Gibberella xylarioides*), which is endemic to soils of Africa. The disease can attack all stages of coffee growth, causing symptoms including wilting, defoliation, necrosis in wood below the bark, and eventually death in infected plants^5^. It produces asexual conidia in dead leaves and ascospores in root and stem bark of infected plants; then the spores germinate into mycelium under appropriate conditions and infect healthy plants, entering vascular bundles via the roots^12^. The nature of infection inside vascular bundles makes the disease difficult to control and treat chemically, with up to 80% loss in production of plantations and sometimes complete loss in smallholdings in the absence of treatments^10,13^. CWD is currently restricted to central and eastern Africa, but the fungal pathogen would be capable of infecting coffee varieties grown in the Americas and in Southeast Asia if transportation occurred^14^. Therefore, it is important that phytosanitary measures ensure CWD is not accidentally introduced to other coffee-growing regions of the world.

**Figure 1:**
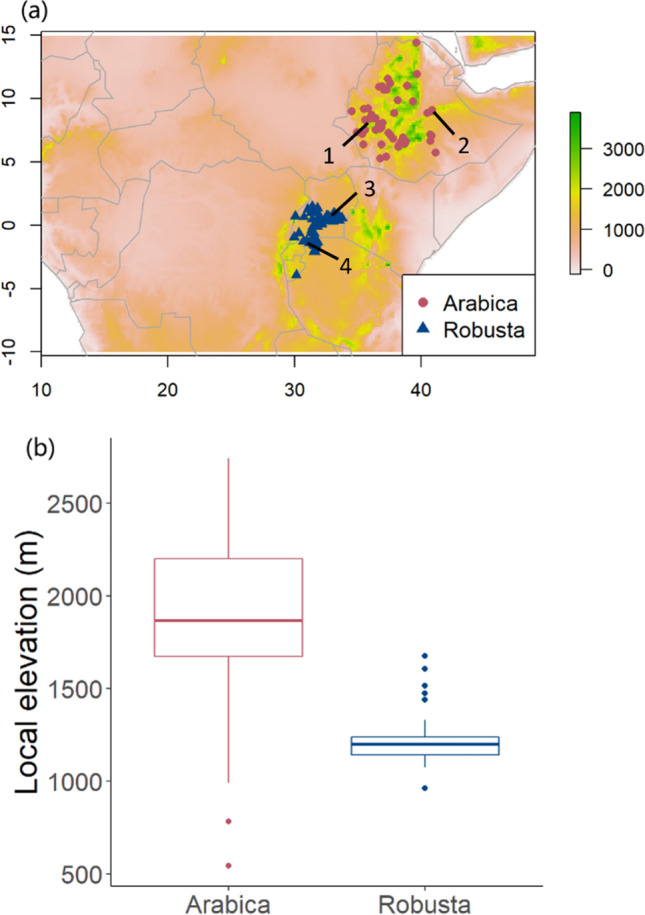
(**a**) A topographic map for elevations around the Ethiopian Highlands and the Congo Basin, with colour of the map indicating elevation. Red circles represent the 51 villages with recorded incidences of the arabica population of CWD, and blue triangles represent the 60 villages with recorded incidences of the robusta population by Oduor et al.^17^. Black numbers and lines show the localities of the four isolates used for growth assays. Isolates specialised to arabica coffee (Coffea arabica): 1 = arabica isolate 389,563, 2 = arabica isolate 393,593. Isolates specialised on ‘robusta’ coffee (C. canephora): 3 = robusta isolate 392,263, 4 = robusta isolate 392,278. Isolate numbers refer to IMI (International Mycological Institute) numbers in the CABI culture collection. (**b**) A boxplot for local elevation of villages with incidences of CWD surveyed by Oduor et al.^17^, illustrating higher elevation for villages with the arabica population of CWD.

CWD first emerged in west and central Africa in the 1920s, with *C. excelsa* and *C. canephora* as major hosts, but it was successfully controlled by improved sanitation and management schemes by the 1960s^10^. However, the disease re-emerged in the 1970s on robusta coffee and spread rapidly in the 1990s and 2000s^16^. The fungus currently has two host-specific populations (Fig. 1a). One specialises on robusta coffee and mainly occurs around the Congo Basin, including the Democratic Republic of the Congo, Uganda, and Tanzania. The other attacks arabica coffee and is only found in Ethiopia, where it was first observed in 1957^5,16^. The robusta variant in Uganda and Tanzania had substantially higher severity (percentage of infected coffee trees per farm) than the arabica variant in Ethiopia^13,17^.

There are several potential explanations for different severity between the host-specific populations, including genetic differences across the fungal strains in their virulence factors^18^, differences in coffee farming practices between different countries (e.g. around half of Ethiopian coffee is harvested from wild and semi-wild coffee groves with high genetic diversity, as well as around 10% from large-scale plantations; whereas coffee from Uganda, Tanzania and the Democratic Republic of Congo is grown in small-scale coffee farms less than 5 hectares in size^14,19^), and varied efficiency of transmission under different temperature and precipitation patterns^20^. Ethiopia lies at a much higher altitude and has a distinct climate compared to tropical lowland countries in the Congo Basin where robusta strains are dominant^5^. Arabica coffee production also favours higher elevation (above 1400 m) to reduce the pressure of coffee leaf rust, while robusta coffee is mainly cultivated at lower altitudes (between 800 and 1500 m, Fig. 1b)^14,17^. This difference in elevation is associated with lower temperature in localities of arabica strains, which can also limit growth and transmission of the fungus, and perhaps explain in part the lower severity^20^.

The effect of temperature on *F. xylarioides* life cycles has not been investigated previously. Studies of other *Fusarium* species on different crops have shown temperature has a significant impact on growth and reproduction of the fungus, however. Cruz et al. observed faster radial growth of 14 *F. oxysporum* isolates from soybean (FO36-FO49) on agar plates between 25 and 30 °C than at lower temperatures^21^, and this finding matched disease severity in soybean seedlings inoculated at different temperatures. Similarly, Manstretta & Rossi found that spore maturation of *F. graminearum* strains was restricted to a temperature of between 20 and 25 °C^22^. Past field observations have also shown CWD has higher incidence rates in warmer years^14^, which coincides with observations in other *Fusarium* species. Temperature is predicted to increase by 2 to 6 °C in most coffee-growing regions of Africa by 2080^23^. This could potentially facilitate the spread of the disease into previously CWD-free regions^1^.

We hypothesize that the two host-specific populations of CWD have different responses to temperature, given the distinct geographical distributions of the two^16^. Specifically, we predict that they should differ in optimal temperature (T_opt_, reflecting adaptation to average temperature of local climates) and critical thermal minimum (CT_min_, reflecting extreme low temperatures of local climates)^24^. If this is true, management strategies of arabica and robusta coffee might need to be assessed separately and adapted to maximise their efficacy against different CWD infections. Thus, to adapt cultivation and management strategies for coffee production, and cope with potential damage caused by climate change, it is necessary to understand the impacts of temperature on the growth and reproduction of strains from each *F. xylarioides* host-specific population.

Here, we investigate the effects of temperature on the distribution, growth and reproduction of *F. xylarioides* using a combination of environmental modelling and growth assays in the laboratory. Firstly, we test whether temperature predicts the distribution and severity of *F. xylarioides* infection using geographical records on severity, measured as the proportions of trees infected per farm, as a measure of disease intensity and of coffee plant loss^17^, and whether severity is predicted to increase with projected future climate change. Secondly, we quantify thermal performance curves of *F. xylarioides* isolates from arabica and robusta populations in vitro in the laboratory. Specifically, we test the hypothesis that the arabica population of *F. xylarioides* grows and reproduces better than the robusta population at lower temperatures, as it is adapted to the colder montane climate in the Ethiopian Highlands. We use mycelial growth, spore production and germination in vitro under a range of temperatures in the laboratory as an assay of thermal performance of growth and reproduction, of the fungus. We could not practically grow coffee plants at this range of temperatures in the laboratory to perform the assays in vivo, but we test whether evidence for thermal dependence of growth and sporulation in vitro reflects climatic correlates of distribution and severity in the field^12^. Together, the results help explain past differences in severity as well as inform predictions for how future climate change might affect the distribution and severity of disease caused by the two host-specific populations.

## Materials and methods

### Climatic modelling

#### Severity data

Severity data were extracted from the CWD survey report in Ethiopia, Uganda, and Tanzania as the proportion of trees infected by *F. xylarioides* per farm^17^. The mean latitude and longitude of regions/villages were extracted using ElevationMap^25^. Incidences in Ethiopia were assigned as the arabica population (n = 51), and those in Uganda and Tanzania as the robusta population (n = 60), as stated by Oduor et al.^17^ (Fig. 1a). The location and severity records of CWD were loaded using R packages ‘readr’ and ‘dismo’^26,27^.

#### Climate data

Bioclimatic variables, which are measures of global temperature and precipitation patterns from 1970 to 2000, were downloaded from the WorldClim database at a spatial resolution of 10 min^23^. As samples of CWD severity were taken during 2002^17^, we extracted data for 2002 from the WorldClim database and calculated bioclimatic variables for this year^28^ to improve accuracy of prediction. CMIP6-projected bioclimatic variables from 2060 to 2080 were also downloaded at a spatial resolution of 10 min^29^. We chose the shared socioeconomic pathway of SSP2-4.5 using predictions of the HadGEM3-GC3.1 model for the CMIP6 data, which represent the intermediate scenario of projected future climate change^29^. The data of global bioclimatic variables were downloaded and transformed for data analysis using R packages ‘dismo’ and ‘raster’^27,30^.

#### Statistical modelling

To assess the effect of temperature on severity of CWD, we extracted bioclimatic temperature variables of villages with incidences of the disease in 2002, using the latitude and longitude data in Oduor et al.^17^, with R package ‘raster’^30^. We expected the transmission of CWD to have a non-linear thermal response, like other fungal plant diseases^31^. Thus, we fitted the severity of arabica and robusta population of *F. xylarioides* as response variables using the Sharpe-Schoolfield equation^32,33^, which is derived from the thermal sensitivity of biochemical reactions and has been widely used for thermal performance curves^34^. This model fits the functional response of trait *R* (in this case severity) to temperature as

$$R\left( T \right) = R_{ref} \frac{{e^{{\left[ {\frac{E}{k}\left( {\frac{1}{{T_{ref} }} - \frac{1}{{\left( {T + 273.15} \right)}}} \right)} \right]}} }}{{1 + e^{{\left[ {\frac{{E_{D} }}{k}\left( {\frac{1}{{\left( {T_{pk} + 273.15} \right)}} - \frac{1}{{\left( {T + 273.15} \right)}}} \right)} \right]}} }}$$

where *R*_*ref*_ is the trait value at a standard temperature *T*_*ref*_, *E* is the activation energy (eV), which describes how quickly performance drops at temperatures below the optimum, *E*_*D*_ is the high temperature de-activation energy (eV), which describes how quickly performance drops at temperatures above the optimum, *T*_*h*_ is the temperature where half of the enzyme units underlying the trait are assumed to be inactive, *T* is the temperature in ºC and *k* is the Boltzmann constant (= 8.62 × 10^–5^ eV*/K*). We used bio1 (annual mean temperature), bio5 (highest temperature of hottest month), and bio6 (lowest temperature of coldest month) as explanatory variables in the models to investigate the response of CWD transmission to average and extreme temperatures respectively. The model was fitted using non-linear least squares (NLS) with the ‘sharpeschoolhigh_1981’ function R packages ‘rTPC’, ‘nls.multstart’ and ‘broom’^32,33^ and using the ‘get_start_vals’ function to obtain sensible starting values. The fitted models estimated the optimum temperature (*T*_*opt*_), severity at optimum temperature (*R*_*max*_), and critical thermal minimum (*CT*_*min*_) and maximum (*CT*_*max*_) of the populations*.*

#### Predicting potential severity in other regions and into the future

We used the fitted models to predict potential severity of arabica and robusta populations across all African coffee-producing countries using WorldClim (1970–2000) bioclimatic data, to reflect the period leading up to the original survey by Oduor et al.^17^. We predicted potential future severity under projected climate changes for the region using CMIP6 (2060–2080) bioclimatic data by predicting values for the model parameters but fed with future temperatures instead of past ones. As temperature will not be the only variable affecting the severity of CWD, we also reported models fitting additional bioclim variables to incorporate precipitation and other seasonal and daily variables (Supplementary Information S1), although our focus is primarily on temperature here.

### Growth assays

#### Isolate selection

All isolates used in the experiments were provided by the CABI-IMI culture collection. The isolates used in this study were accessed and used in full compliance with UK Regulation implementing the Nagoya Protocol on Access and Benefit Sharing https://www.gov.uk/guidance/abs of the Convention on Biodiversity. Four *F. xylarioides* isolates, two from the arabica and two from the robusta populations, were chosen from locations with typical montane and tropical climatic patterns respectively (Fig. 1a). Specifically, average monthly temperature and precipitation of each location was extracted from the WorldClim database^23^ and cross-referenced to pick isolates from locations spanning the range of monthly temperatures and precipitations observed for that host population. Isolates were used for growth assays and spore production and germination measurements. (Table S1).

#### Temperature selection

Based on the current and projected range of temperatures experienced using WorldClim and CMIP6 records^23,29^, we conducted growth assays at 10, 15, 20, 25, 30, 35, and 40 °C to analyse response in growth and sporulation rates. Three replicates of each *F. xylarioides* isolate was grown at each temperature to measure variation in responses. Due to the limited availability of incubators, growth assays were performed in 2 batches. The first round included 15, 20, 25, 30, and 40 °C, while the second covered 10, 15, 20, 25, 30 and 35 °C to ensure thermal performance in growth and reproduction of isolates were captured under a range of temperatures. We conducted the first batch of growth assays from 20th to 31st January 2021, and the second batch from 7 to 18th May 2021. Note that the 40 °C and 35 °C treatments were only conducted in batch 1 and 2, respectively, 10 °C was also conducted only in batch 2, whereas all other treatments were repeated in both batches.

#### Growth assays

Assays were performed on 120 mm synthetic nutrient agar (SNA) plates based on the protocol of Halder^35^. A block of agar, approximately 3 mm × 3 mm × 3 mm, was transferred from the active edge of fungal cultures (stock plates); these stock plates contained mycelium of the corresponding isolate and inocula were placed at the centre of a fresh plate using a sterile disposable loop. A fine-tip marker was used to trace the outline of the agar block on the base of each 60mm Petri dish. The plates were grown at 25 °C for 48 h, after which the new colony outline was drawn in a different colour and photographed. Plates were then randomly assigned to and grown at the range of temperatures for 9 days (216 h, 3 replicates for each strain x temperature x batch combination). Photographs were taken of each plate every 24 h and analysed using Photoshop (Adobe Photoshop CC 2015.5) and ImageJ (ImageJ 1.53c) to count the pixel numbers of the fungal colony and the plate. The growth rate of the colony was then calculated using the following equations:

$$Colony\;area = 3600\pi \times \frac{Colony\;pixel\;number}{{Plate\;pixel\;number}} \left( {{\text{mm}}^{2} } \right)$$

$$Growth\;rate = \frac{{ \sqrt {Colony\;area} }}{\pi \times Number\;of\;days} \left( {{\text{mm}}\;{\text{day}}^{ - 1} } \right)$$

Average daily growth rate between days four and eight was used to represent growth rates, reflecting the period of maximum growth after an initial lag and before a final slow down.

#### Spore counts

After the last sampling from the growth assay was completed, fungal spores were harvested from two out of three replicates per isolate per temperature, by pouring 10 mL distilled water and 1 droplet of 0.05% tween-80 (polysorbate 80) solution onto the mycelia and scraping its surface to release spores from the colony. Spore concentration was measured for each sample using a haemocytometer, with the average spore concentration in the plate calculated following BTI protocol^36^. Each of the two replicates from each isolate per temperature was sampled twice, with 1 measurement for each of 10 haemocytometer chambers. This gave a total of 20 repeated measurements of spore concentration for each replicate, which were averaged. We then calculated sporulation rate as the number of spores produced per mm^2^ of mycelium by dividing the average spore concentration × 10 mL by the area of the mycelium for that sample. This measure aimed to account for differential spore production rate due to varied sizes of colonies.

#### Spore germination rate

This was measured by spreading a thin line of inoculum produced from the last section in a fresh SNA plate. Each combination of strain × temperature × batch had 1 plate sampled. The plates were kept at room temperature for 24 h to allow the spores to germinate and then viewed under a compound microscope (Meiji ML2000 microscope) with × 4 magnification. The total number of spores in the sight and the number of germinated spores with hyphae growing out were recorded. Counts were made from 3 different points on each plate. Germination rate was calculated as the number of germinated spores divided by the total number of spores, averaged across the 3 points measured on each plate.

#### Statistical analyses

We used a linear mixed-effects model (LMM), with average daily growth rate as the response variable, and factorised temperature, host-specific population and their interactions as fixed effects, and batch and strain as random effects, using R packages ‘lme4’ and ‘lmerTest’^37,38^. By analysing temperature as factors, we could compare nonlinear responses to temperature within and between populations. Pairwise comparisons between mean growth rates at different temperatures were conducted with Tukey adjustments using R package ‘emmeans’^39^. The same approach was then repeated for sporulation rates and spore germination rates as response variables in turn. A NLS model was also fitted to the three response variables in turn (growth rate, sporulation rate, germination rate) at each incubation temperature, following the Sharpe-Schoolfield model (high-temperature inactivation only) as described above for climate data. We estimated *T*_*opt*_*, R*_*max*_*, CT*_*max*_ and *CT*_*min*_ for each response variable in turn. Code and data for all statistical analyses is available at *github*.

## Results

### Climatic modelling of the thermal dependence of severity of *Fusarium xylarioides*

#### Response to mean annual temperature

**Figure 2:**
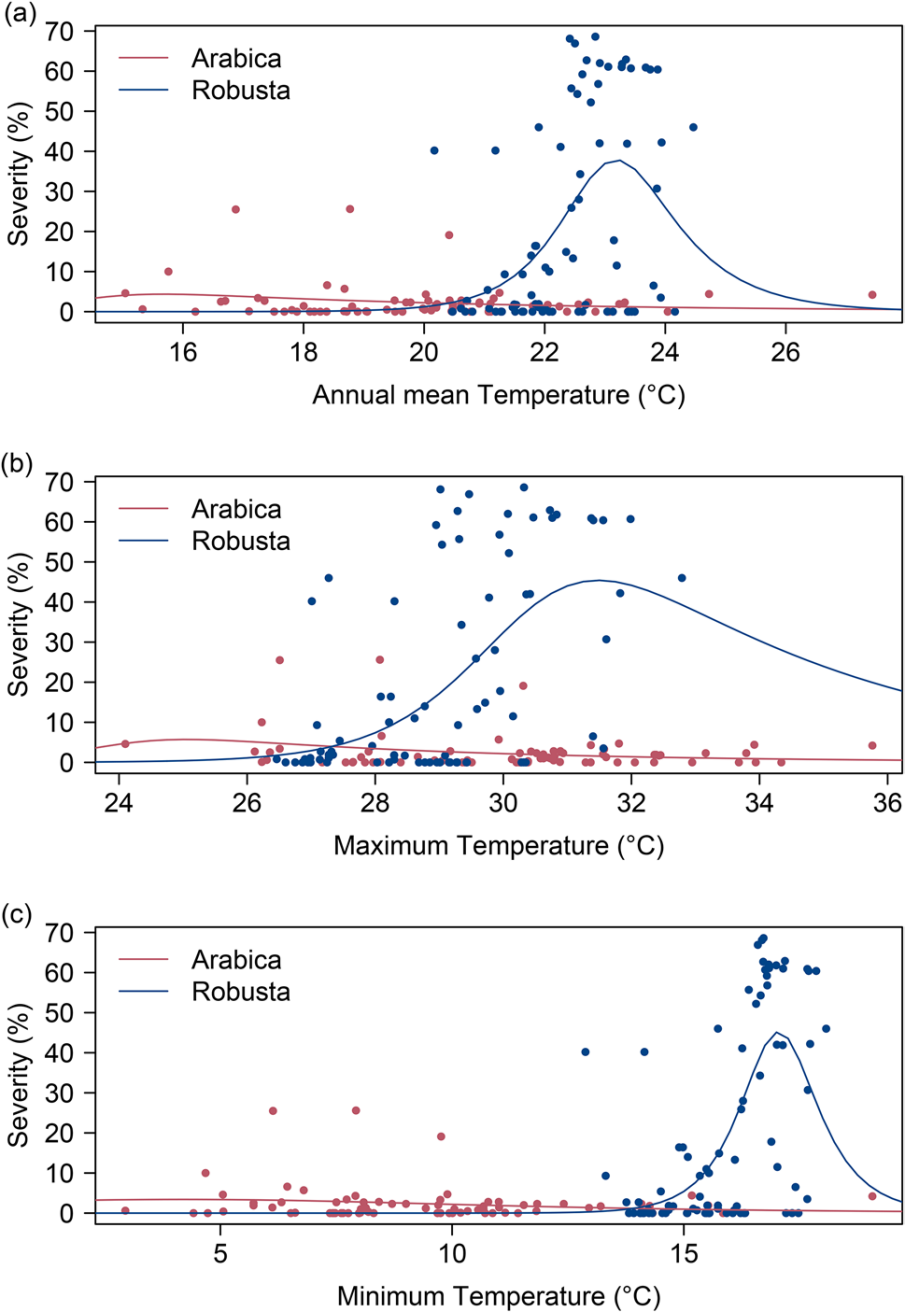
Predicted response in severity (percentage of trees infected per farm) of *F. xylarioides* populations to mean and extreme temperatures. The circles represent the mean severity of each sampled village. The smooth lines represent the thermal performance in severity of *F. xylarioides* populations predicted by Sharpes-Schoolfield non-linear least squared models. (**a**) Response *F. xylarioides* severity to annual mean temperature. (**b**) Response *F. xylarioides* severity to maximum temperature. (**c**) Response *F. xylarioides* severity to minimum temperature.

The non-linear least squared (NLS) model fitted a flat thermal response curve for the arabica host-specific populations, with low severity across a wider range of temperatures. In contrast, the severity of the robusta host-specific population of *F. xylarioides* showed a strong bell-shaped response to mean annual temperatures, with higher optimum mean temperatures and maximum severities for the than the arabica form(Fig. 2). The arabica population was estimated to have maximum severity (*R*_*max*_*)* of 4.37% (95% confidence limits: 2.68–9.00%) at mean temperature of 15.7 °C (95% CL 15.0–22.7 °C), and critical thermal minimum (*CT*_*min*_) and maximum (*CT*_*max*_) at 12.0 °C (95% CL -Inf-14.7 °C) and 33.5 °C (95% CL 21.0–51.3 °C). The robusta population was estimated to have maximum severity of 37.86% (95% CL 29.8–46.3%) at mean temperature of 23.2 °C (95% CL 22.9–24.2 °C) with *CT*_*min*_ of 20.3 °C (95% CL 18.4–20.6 °C) and *CT*_*max*_ of 26.6 °C (95% CL 25.6–44.2 °C). The model predictions also showed the arabica population displays higher severity than the robusta population below approximately 20.5 °C, while the latter has higher severity above this mean temperature (Fig. 2a). The only cases with severity above 10% for arabica were in the lower half of the observed temperature range.

#### Response to extreme temperatures

NLS models for extreme temperatures also predicted the arabica population to have lower optimum temperature, with better tolerance to coldness and higher severity at low temperatures. The models for maximum temperature suggested the severity of the arabica population to be highest (5.72%, 95% CL 2.50–14.44%) at maximum temperature of 25.1 °C (95% CL 24.1–29.6 °C), and CT_max_ of 39.4 °C (95% CL 30.5–52.2 °C). Meanwhile, the robusta population severity was highest (54.41%, 95% CL 38.3–77.5%) at maximum temperature of 31.5 °C (95% CL 30.7–32.8 °C), with CT_max_ of 44.9 °C (95% CL 33.6 °C-Inf). Despite the robusta population having much higher peak severity, the arabica population is predicted to have higher severity than the former at locations with maximum temperature below approximately 27.2 °C (Fig. 2b).

The minimum temperature models also revealed a similar difference between the populations. Severity of the arabica population was predicted to peak (3.41%, 95% CL 2.11–14.10%) at minimum temperature of 4.2 °C (95% CL 3.0 °C-16.2 °C), with CT_min_ outside the range of minimum temperatures across sampled sides (95% CL -Inf-5.7 °C). In contrast, peak severity of the robusta population of 45.2% (95% CL 36.6–51.0%) was predicted at minimum temperature of 17.1 °C (95% CL 16.9–17.5 °C), with CT_min_ at 14.36 °C (95% CL 12.7–14.5 °C). The arabica population is predicted to have higher severity than robusta below minimum annual temperature of 14 °C (Fig. 2c).

### Spatial predictions of severity of *Fusarium xylarioides*

#### Past severity

**Figure 3:**
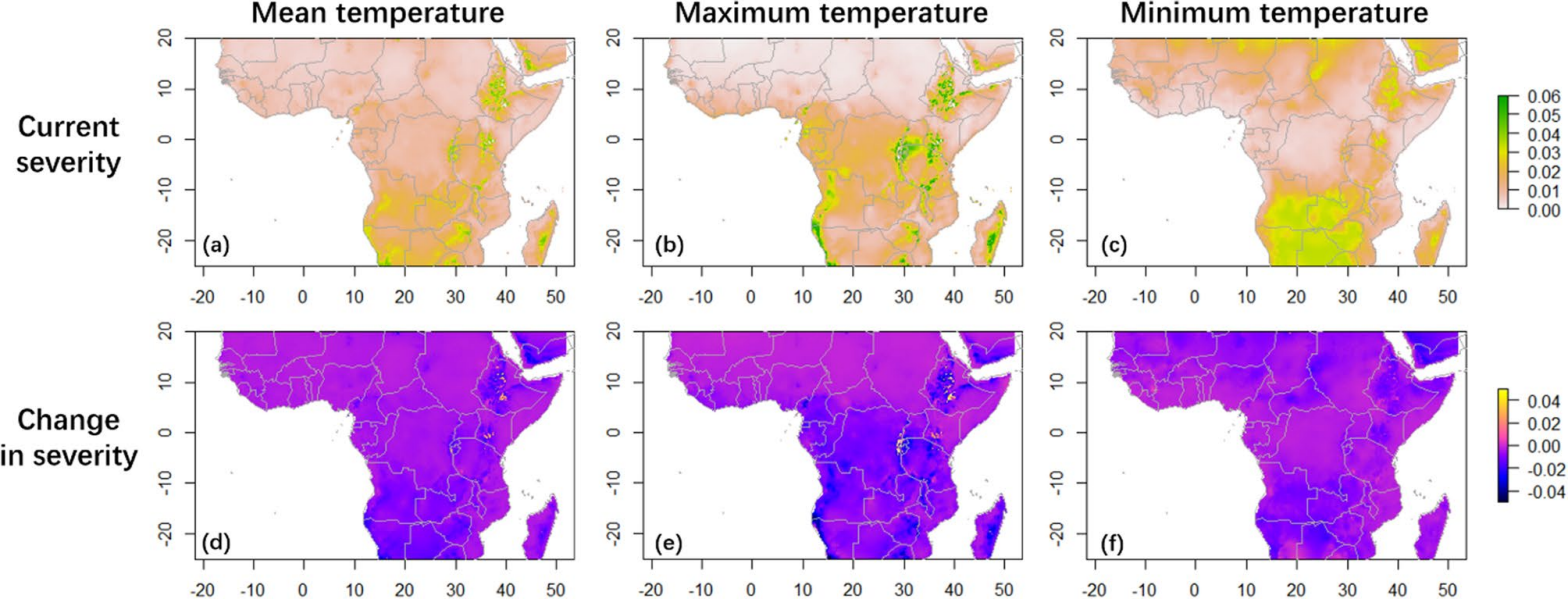
Predicted severity for the arabica population of *F. xylarioides* in major coffee-growing countries of Africa. Negative values in (d)(e)(f) indicate a decrease in severity. (**a**) Predicted severity under mean temperature of past climatic data (1970–2000) (mean = 1.12%). (**b**) Predicted severity under maximum temperature of past climatic data (mean = 1.14%). (**c**) Predicted severity under minimum temperature of past climatic data (mean = 1.40%). (**d**) Change in mean-temperature predictions of severity between past and future (2060–2080) climatic data (mean change = − 0.52%). (**e**) Change in maximum-temperature predictions of severity between past and future climatic data (mean change = 0.61%). (**f**) Change in minimum-temperature predictions of severity between past and future climatic data (mean change = − 0.53%).

WorldClim (1970–2000) data were used to extrapolate the models reported above to predict past spatial variation in severity across coffee-producing regions of Africa (between 25°S and 20°N, distribution of coffee producing countries can be found in Fig. S1) based on temperature alone. The arabica population is confined to Ethiopia, and its predicted severity is, as expected, also low across Africa under 1970–2000 climate for all three measures of temperature (mean temperature model: mean = 1.12%, SD = 0.690%; maximum temperature model: mean = 1.14%, SD = 0.996%; minimum temperature model: mean = 1.40%, SD = 0.914%). Some coffee-farming regions were shown to have higher predicted severity than other regions (over 4%), however, including the Ethiopian mountains (the current distribution of the arabica population), Burundi, Rwanda, southern Kenya, and Zimbabwe (all regarded as important arabica coffee-producing regions by ICO^40^ (Fig. 3a,b,c). The mean and maximum temperature models also projected Tanzania as a suitable habitat for the arabica isolates (Fig. 3a,b), whereas the minimum temperature model included western Zambia (Fig. 3c). Although not a major producer of arabica coffee, the models also predict Angola and northern South Africa to be suitable habitats of the arabica population (with predicted severity > 4%, Fig. 3a,b,c).

**Figure 4:**
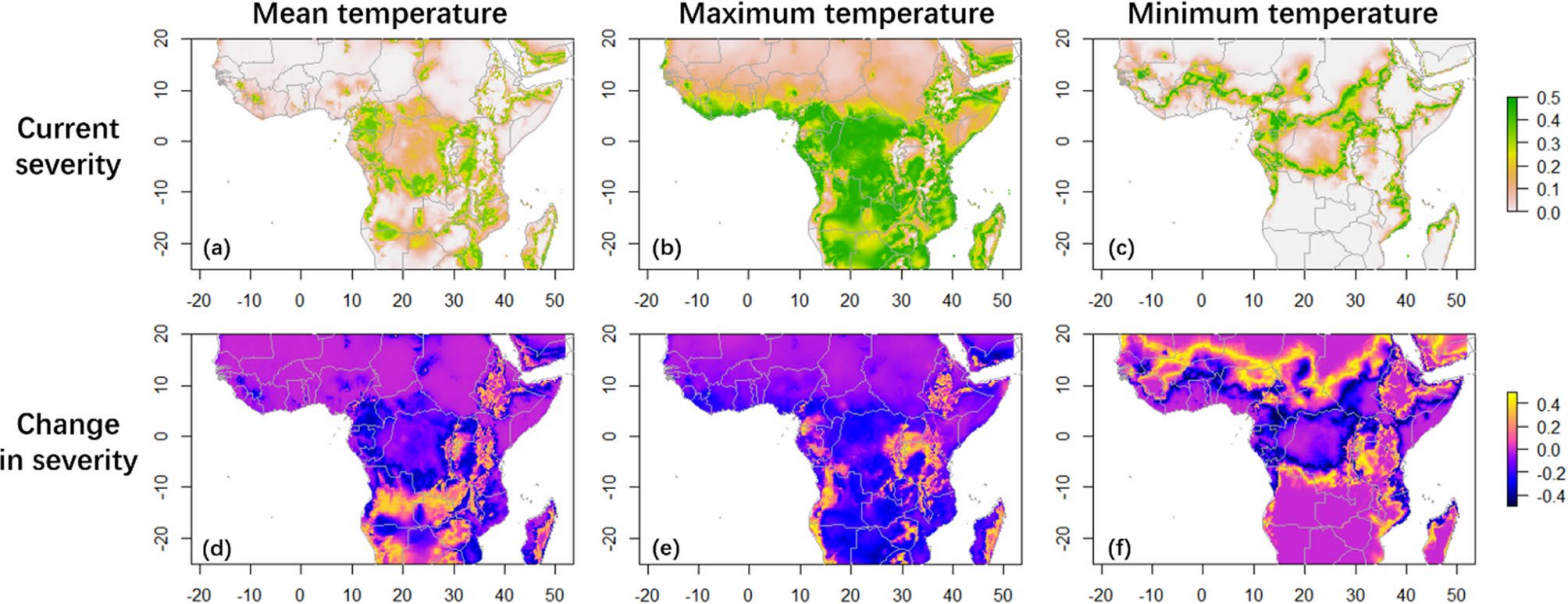
Predicted severity for the robusta population of *F. xylarioides* in major coffee-growing countries of Africa. Negative values in (d)(e)(f) indicate a decrease in severity. (**a**) Predicted severity under mean temperature of past climatic data (1970–2000) (mean = 8.89%). (**b**) Predicted severity under maximum temperature of past climatic data (mean = 23.52%). (**c**) Predicted severity under minimum temperature of past climatic data (mean = 7.86%). (**d**) Change in mean-temperature predictions of severity between past and future (2060–2080) climatic data (mean change = − 4.51%). (**e**) Change in maximum-temperature predictions of severity between past and future climatic data (mean change = -8.73%). (**f**) Change in minimum-temperature predictions of severity between past and future climatic data (mean change = 0.52%).

All three models for the robusta population predicted on average higher severity compared to the corresponding models of the arabica population (mean temperature model: mean = 8.89%, SD = 11.172%; maximum temperature model: mean = 23.52%, SD = 15.256%; minimum temperature model: mean = 7.86%, SD = 12.642%). The robusta coffee-growing regions predicted to have high severity (> 30%) include northern Tanzania, the Congo Basin (including Uganda, Democratic Republic of the Congo, Central African Republic, and Republic of the Congo), Cameroon, west Africa (including Côte d'Ivoire, Gabon and Guinea), and Nigeria (Fig. 4a,b,c)^14,41^. Furthermore, the maximum-temperature model predicted most regions south of 10°N to have high severity (Fig. 4b). Though Malawi, southern Ethiopia and Madagascar are not major producers of robusta coffee, these countries were also estimated to be climatically favourable for high severity of the robusta population by all three models (Fig. 4a,b,c).

#### Future severity

CMIP6 (2060–2080) bioclimatic data were used to extrapolate the models to predict future potential severity across coffee-producing regions of Africa based on future temperature projections, assuming correlation of severity with temperature variables remained unchanged. All three models predicted a significant near-half reduction in severity of the arabica population under projected 2060–2080 temperature changes (Welch Two Sample t-test, mean temperature model: mean change = − 0.52%, t_108,089_ = − 165.41; maximum temperature model: mean change = − 0.61%, t_106,150_ = − 137.36; minimum temperature model: mean change = − 0.53%, t_126,142_ = − 114.72. *P*-value < 0.001 for all three models). However, some regions near Bale Mountains, Simien Mountains and Ethiopian Highlands were shown to experience an increase in severity of CWD by all models, despite the overall decrease in severity in this region (> 2%, Fig. 3d,e,f). In addition, arabica population severity was predicted to increase in some locations of south Kenya, Rwanda, Burundi and east DRC, by the mean and maximum temperature models (Fig. 3d,e).

The mean and maximum-temperature models also predicted a near-half reduction in severity of the robusta population under 2060–2080 climate, while the minimum-temperature model predicted a slight increase (Welch Two Sample t-test, mean temperature model: mean change = − 4.51%, t_128,589_ = − 79.30; maximum temperature model: mean change = − 8.73%, t_127,005_ = − 117.01; minimum temperature model: mean change = 0.52%, t_131,816_ = 6.96. *P*-value < 0.001 for all three models). However, in all models, some regions would experience a large increase in severity (> 20%), and the major robusta-coffee-producing areas subjected to this predicted increase were east Democratic Republic of the Congo, Tanzania, and Uganda (Fig. 4d,e,f)^40^. Angola and Madagascar also produce some robusta coffee^41^, and were predicted to have increased severity (Fig. 4d,e). Rwanda and Burundi were predicted to experience increased severity, but they are currently arabica-producing regions. As context for our temperature-only models, climate models incorporating additional bioclimatic variables yielded similar average levels for net changes in severity by 2060–2080, but the exact regions projected to experience increased severity were different from the temperature-only models for both host-specific populations (Supplementary Information S1, Fig. S2).

### Growth assays in the laboratory

#### Mycelial growth rates

**Figure 5:**
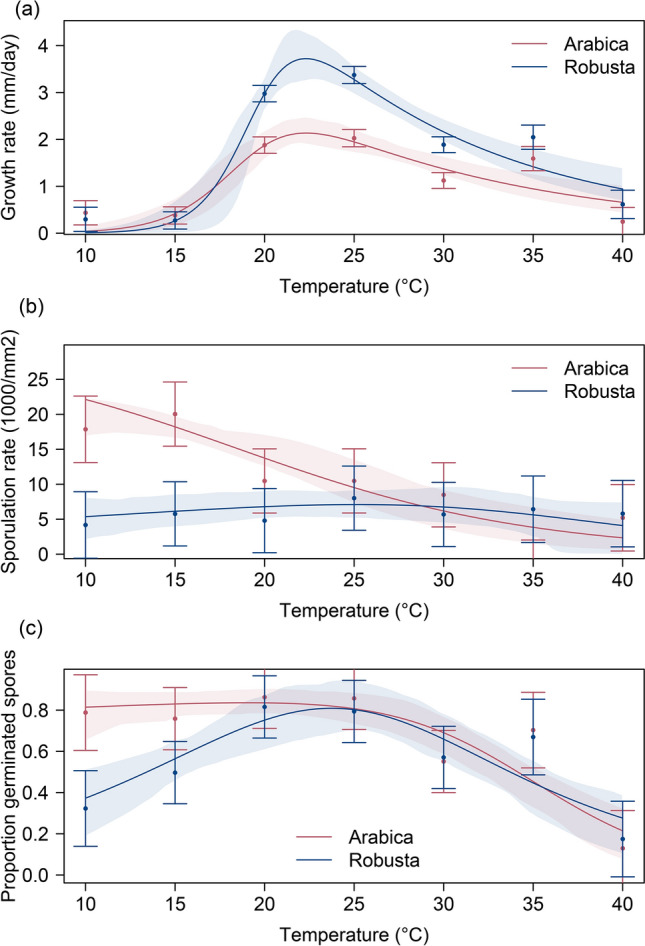
Predicted thermal response in growth and reproduction characteristics of *F. xylarioides* populations. The dots and error bars represent the mean spore density and standard error predicted by linear mixed-effect model. The smooth lines represent the thermal performance of *F. xylarioides* populations predicted by Sharpes-Schoolfield non-linear least squared models, with shaded regions indicating 95% confidence limits of the predictions. (**a**) Response of *F. xylarioides* mycelium growth rates to temperature. (**b**) Response of *F. xylarioides* sporulation rates to temperature. (**c**) Response of *F. xylarioides* spore germination rates to temperature.

The linear mixed-effect model accounted for a large proportion of variations in growth rates in replicates across two *F. xylarioides* isolates of each host-specific population (model adjusted R^2^ = 0.777), and the fixed effects accounted for most of variation (fixed effects adjusted R^2^ = 0.771). There was a significant interaction between host and temperature (*F*_*6,102*_ = 4.9, *P* < 0.001, Fig. 5a, Table S2) and the growth rates of the robusta population significantly exceeded the arabica population at 20 °C (pairwise comparisons Tukey HSD t_21.2_ = 4.43, *P* < 0.05) and 25 °C (Tukey HSD t_24.5_ = 5.23, *P* < 0.01), while differences were not significant at remaining temperatures (Tukey HSD: 10 °C: t_47.8_ = − 0.43; 15 °C: t_24.5_ = − 0.41; 30 °C: t_18.7_ = 3.21; 35 °C: t_47.8_ = 1.40; 40 °C: t_69.7_ = 0.94. *P* > 0.05 for temperatures mentioned). Contrary to our prediction that arabica isolates should grow significantly faster at low temperatures, isolates of both populations showed a similar shape of thermal response: slow growth rates at 10 °C and 15 °C, a steep increase at both 20 °C and 25 °C, and a slower decline at 30 °C and above (model outputs in Table S2). The Sharp-Schoolfield model predicted an optimum temperature for growth of arabica strains at 22.3 °C (95% CL 20.7–23.6) with maximum growth rate of 2.14 mm/day (95% CL 1.9–2.5) and *CT*_*min*_ at 12.0 °C (95% CL 8.2–15.0, Fig. 5a). The robusta population was also predicted to grow fastest at 22.3 °C (95% CL 21.5–22.9) with growth rate of 3.72 mm/day (95% CL 3.3–4.4) and *CT*_*min*_ at 14.5 °C (95% CL 11.5–17.4). *CT*_*max*_ in both cases was extrapolated well outside experimental temperatures (> 50 °C) and hence not reported. The fitted model predicted that arabica isolates grow slightly faster than robusta isolates below 17 °C, but this difference was not significant in the LME model.

#### Sporulation rates

Our linear mixed-effect model explained most of the variation in unit spore concentrations of *F. xylarioides* isolates in general (model adjusted R^2^ = 0.818), with the fixed effects of temperature and host-specific population accounting for just over half (adjusted R^2^ = 0.384). There was a significant interaction between host population and temperature (*F*_*6,71*_ = 15.4, *P* < 0.001, Fig. 5b, Table S3): the arabica strains displayed greater sporulation rate at 15ºC and below than at warmer temperatures (pairwise comparisons Tukey HSD: 10 °C: t_47.8_ = − 0.43; 15 °C: t_24.5_ = − 0.41. *P* < 0.05 at both temperatures), whereas the robusta population showed no trend in sporulation with temperature (LME model with just robusta data and temperature as a fixed effect, *F*_*6,35*_ = 1.03, *P* = 0.42, Fig. 5b). The Sharp-Schoolfield model predicted an optimum temperature for sporulation of arabica strains at 10 °C (95% CL 10.0–13.8) with maximum sporulation rate of 22.1 × 10^3^ spores/mm^2^ of mycelium (95% CL 17.4–23.8 × 10^3^, Fig. 5b). The Sharp-Schoolfield model estimated a higher optimum temperature for the robusta population, but in line with the LME model it failed to fit a well-defined line (optimum temperature 25.1ºC, 95% CL 10–40; R_max_ 7.1 × 10^3^ spores/mm^2^, 95% CL 6.0–11.0 × 10^3^).

#### Spore germination rates

The linear mixed-effects model explained most of the variation in spore germination rates of *F. xylarioides* isolates (model adjusted R^2^ = 0.725), and temperature and population accounted for most of the variation (fixed effects adjusted R^2^ = 0.512). Due to lower replication for the germination assay, the interaction between host population and temperature was not significant (*F*_*6,27*_1.1, *P* = 0.39, Fig. 5c, Table S4) but the pattern was qualitatively similar to sporulation rate: the populations showed similar germination rates at higher temperatures, but arabica strains had higher germination rates than robusta strains at lower temperatures (Fig. 5c). The NLS Sharp-Schoolfield model predicted a flat curve for the arabica population strains with optimum germination temperature of 19.1 °C (95% CL 10.0–24.7) and maximum germination rate of 0.84 (95% CL 0.79–0.94). The robusta population was predicted to have a slightly higher optimum temperature (23.8ºC, 95% CL 20.7–26.6) and similar maximum germination rate (0.81, 95% CL 0.70–0.93), but a steeper fall in germination rate at lower temperatures (estimated activation energy parameter *e* = 0.57 for robusta strains compared to *e* = − 0.23 for arabica strains, 95% CI do not overlap below 15 °C, Fig. 5c).

## Discussion

In both climate modelling and growth assays, the two host-specific populations of *F. xylarioides* showed distinct responses to temperature that reflect their elevational distributions. The NLS Sharp-Schoolfield models imply the optimum temperatures (*T*_*opt*_) for severity in nature (based on mean annual temperature), hyphal growth, sporulation (weakly) and spore germination of the robusta population were all clustered around 23 °C, and their thermal response were roughly bell-shaped around *T*_*opt*_. In contrast, while the hyphal growth rate of the arabica population displayed a similar thermal performance profile and *T*_*opt*_ to robusta (which also corresponds to previously measured *T*_*opt*_ for growth in other *Fusarium* species ranging from 16 to 27 °C^21,42^), all other measures displayed higher relative performance for arabica isolates at lower temperatures. In climate models, the arabica population displayed a lower inferred *T*_*opt*_ and higher relative severity at lower temperatures. In the growth assays, sporulation and germination of arabica isolates occurred at a higher rate at low temperatures (10 °C and 15 °C) than robusta isolates. These findings support the hypothesis that the arabica population has higher tolerance to cold conditions observed at higher elevations where arabica coffee is grown^17,23^.

Our results imply that the arabica population is ‘cold adapted’; which is observed in spore production and germination, which show more distinctive thermal responses. Potential explanations for this include accumulation of cryoprotectants^43,44^, alteration in composition of membrane lipids and fatty acids^44^, and evolution of psychrophilic (cold-active) enzymes in sporulating hyphae and spores^45,46^. Gene ontology terms for synthesis of trehalose, a commonly found cryoprotectant in microbes, have been found in the arabica population of *F. xylarioides*^47^, which might support the first explanation. As *F. xylarioides* is a soil-borne pathogen^5^, trehalose could prevent the arabica population from freeze–thaw damage which it potentially experiences in soils of Ethiopian Highlands during the cooler season^23^. Indeed, the arabica isolates had higher growth rates below 17 °C than the robusta isolates, a trend which reversed at warmer temperatures. Hyphal growth rates partly represent the infective abilities of the pathogen under ideal conditions, and could thus indicate a trade-off in virulence for increased resilience in cold environments. On the other hand, *Coffea arabica* is less resilient to diseases than robusta coffee^9^. Thus, arabica strains might need to invest less resources in hyphal growth to infect its target host plant compared to robusta strains. Both explanations suggest differential allocation of resources in growth and reproduction between the two populations, which could be investigated in future.

The phenotypic differences observed in the laboratory suggest that higher severity of coffee wilt disease (CWD) of the robusta population in Uganda and the Democratic Republic of Congo, compared to the arabica population in Ethiopia^17^, is not solely driven by different environmental temperatures across the countries, but also by differential thermal responses in growth and transmission of the two populations. However, growth, sporulation and germination cannot fully represent the life cycle of *F. xylarioides*. Other processes including spore dispersion and colonisation in host coffee plants will also affect its rate of transmission and infection. Therefore, it would be beneficial for future research to investigate the effects of temperature on these processes, especially on infection and growth rates in coffee plants, something we were unable to do. As *F. xylarioides* is soil-borne, future work could model investigate the effects of soil temperature (currently poorly measured at regional scales in Africa^48^).

Through climatic modelling, we have also shown that although *F. xylarioides* is currently restricted to areas around the Congo Basin and Ethiopian Highlands, temperature patterns in several other coffee-growing regions of Africa are also suitable for one or both populations of the fungus. Many countries of these regions are important producers of arabica and robusta coffee, some relying heavily on coffee as a source of foreign currency^10^. These regions, such as west and south Africa (especially Angola, Zimbabwe, South Africa, Cameroon, Côte d'Ivoire, Gabon and Guinea), and Madagascar, could implement a range of measures, such as phytosanitary certificates; training extension workers to connect with smallholder growers; and conducting CWD surveys, to prevent future spread of the disease. These measures would ensure that any future outbreak is rapidly recognised and addressed. Several measures were proven highly effective in controlling the pathogen in Ethiopia and Tanzania, such as uprooting and burning infected coffee plants, phytosanitary certificate control, and education for coffee farmers on relevant information^13,49^, which can be promoted in other coffee-farming regions. Our future climate models predict overall decreases in severity because the mean temperature of modelled regions is predicted to be 27–35 °C by 2060–2080^29^, which is several degrees higher than the estimated optimum temperatures of both populations. Some countries are predicted to have climates favourable to increased severity, especially around the Congo Basin and Ethiopia. In principle, longer-term selection experiments could test whether the fungi are able to adapt to increase in temperatures, which then would alter predictions as the effects of particular temperatures on severity would then also change in the future.

Though we have shown the strong effect of temperature on growth and transmission of both populations of *F. xylarioides*, temperature is not the only determining factor for CWD infections. Earlier research in other *Fusarium* species suggested the production and maturation of spores have a narrow range of humidity requirements^22^. Prior field observations have also shown alternations in rainfall promotes transmission of CWD, which provides the favoured humidity conditions for production of asci, and facilitate the release of ascospores from them^50^. Expanding our models to include other bioclimatic variables led to similar average severity predictions, but the regions projected to be suitable for *F. xylarioides* populations and subjected to future increases in severity were both more concentrated around equatorial Congo Basin (Fig. S2). However, both binomial generalised linear models had low coefficient of determination, especially for the arabica population (Supplementary Information S1), suggesting the need for more data and modelling to explain variation in their severity. Management approaches also strongly affect transmission of CWD. Mulching, herbicides, copper paints and hand-weeding suppress infection, while slashing weeds produces wounds on the plant, which allows more infection of *F. xylarioides*. Any systematic differences between regions or coffee species might further explain differences in severity between regions and host populations and would need taking into account. Thus, although we focused on temperature specifically, future research into how other climatic and agronomic variables affect the ecology of *F. xylarioides* populations and their corresponding coffee hosts would be helpful to predict future risk of disease across coffee-producing regions.

By demonstrating a match between macroecological patterns of disease severity and the thermal profiles growth and reproduction of disease-causing organisms in the laboratory, we provide evidence for temperature adaptation of CWD populations adapted to arabica and robusta coffee. As many parts of the world are predicted to experience warming, a combination of environmental modelling and physiological measurements with culture collection isolates can be a useful general tool to help to predict temperature-dependent life stages. Such information can feed into epidemiological models of future risk of plant diseases in changing climate.

## Supplementary Information

Supplementary Information.

## Data Availability

Datasets and analysis code are available at https://github.com/tim-barra/Fx.

## References

[1] Haggar, J. and Schepp, K. (2012) Coffee and Climate Change: Impacts and Options for Adaptation in Brazil, Guatemala, Tanzania and Vietnam. Climate Change. *Agriculture and Natural Resources Working Paper Series No. 4*. Natural Resources Institute, University of Greenwich, London, 12. Available from: https://www.nri.org/images/documents/development-programmes/climate_change/publications/D5930-11_NRI_Coffee_Climate_Change_WEB.pdf.

[2] Ortiz R (2008). Climate change: Can wheat beat the heat?. Agric. Ecosyst. Environ..

[3] Tubiello FN, Soussana J, Howden SM (2007). Crop and pasture response to climate change. Proc. Natl. Acad. Sci..

[4] Newbery F, Qi A, Fitt BD (2016). Modelling impacts of climate change on arable crop diseases: progress, challenges and applications. Curr. Opin. Plant Biol..

[5] Rutherford MA (2006). Current knowledge of coffee wilt disease, a major constraint to coffee production in Africa. Phytopathology.

[6] Research and Markets (2022) *Global Coffee Market: Analysis By Product Type (Roast & Ground, Soluble and Single Serve), By Coffee Bean Type (Arabica and Robusta), By Region Size and Trends with Impact of COVID-19 and Forecast up to 2027*. Research and Markets. Available from: https://www.researchandmarkets.com/r/em3gwn.

[7] World Trade Organization (WTO). (2023) *WTO STATS.* Available from: https://stats.wto.org/.

[8] United States Department of Agriculture (USDA). (2017) Coffee: World Markets and Trade*. Foreign Agricultural Service.* Available from: https://downloads.usda.library.cornell.edu/usda-esmis/files/m900nt40f/c821gk15t/8336h2335/tropprod-12-15-2017.pdf.

[9] Rubayiza AB, Meurens M (2005). Chemical discrimination of arabica and robusta coffees by Fourier transform Raman spectroscopy. J. Agric. Food Chem..

[10] Mulatu A, Shanko D (2019). Incidence and Prevalence of Coffee wilt Disease (*Gibberella xylarioides*) and Its Impact on the Rural Livelihoods in Western Guji Zone, Southern Ethiopia. Am. J. Biosci..

[11] Al-Abdulkader AM, Al-Namazi AA, Al-Turki TA, Al-Khuraish MM, Al-Dakhil AI (2018). Optimizing coffee cultivation and its impact on economic growth and export earnings of the producing countries: The case of Saudi Arabia. Saudi J. Biol. Sci..

[12] Alemu T (2012). A review of coffee wilt disease, *Gibberella xylarioides* (*Fusarium xylarioides*) in Africa with special reference to Ethiopia. Ethiopian J. Biol. Sci..

[13] Phiri, N. & Baker, P. (2009) *A synthesis of the work of the Regional Coffee Wilt Programme 2000–2007. Coffee wilt disease in Africa.* CABI. https://www.researchgate.net/publication/274389248_Coffee_Wilt_in_Africa_Final_technical_report_of_the_Regional_Coffee_Wilt_Programme_2000-07.

[14] Flood, J. (2009) *Coffee Wilt Disease*. [e-book], CABI. https://www.cabdirect.org/cabdirect/abstract/20103151287.

[15] Girma, A., *et al*. (2007) Tracheomycosis (*Gibberella xylarioides*)-a menace to world coffee production: evidenced by cross inoculation of historical and current strains of the pathogen. *21st International Conference on Coffee Science, Montpellier, France, 11–15 September, 2006*. Association Scientifique Internationale du Café (ASIC). pp.1268–1276. https://www.cabdirect.org/cabdirect/abstract/20073222232.

[16] Olal S (2018). Using translation elongation factor gene to specifically detect and diagnose *Fusarium xylaroides*, a causative agent of coffee wilt disease in Ethiopia, East and Central Africa. J. Plant Pathol. Microbiol..

[17] Oduor, G., et al. (2003) Surveys to assess the extent and impact of coffee wilt disease in East and Central Africa. Final Technical Report. *Surveys to Assess the Extent and Impact of Coffee Wilt Disease in East and Central Africa.Final Technical Report.* https://www.cabdirect.org/cabdirect/abstract/20113101808.

[18] Peck L, Nowell R, Flood J, Ryan M, Barraclough T (2021). Historical genomics reveals the evolutionary mechanisms behind multiple outbreaks of the host-specific coffee wilt pathogen *Fusarium xylarioides*. BMC Genom..

[19] Wassie AK (2019). Integrated diseased management on coffee wilt disease caused by *Fusarium Xylarioides* and its distribution in Ethiopian review. Agric. Res. Technol..

[20] Elderd BD, Reilly JR (2014). Warmer temperatures increase disease transmission and outbreak intensity in a host–pathogen system. J. Anim. Ecol..

[21] Cruz DR, Leandro LF, Munkvold GP (2019). Effects of temperature and pH on *Fusarium oxysporum* and Soybean Seedling Disease. Plant Dis..

[22] Manstretta V, Rossi V (2016). Effects of temperature and moisture on development of *Fusarium graminearum* perithecia in maize stalk residues. Appl. Environ. Microbiol..

[23] Fick SE, Hijmans RJ (2017). WorldClim 2: New 1 km spatial resolution climate surfaces for global land areas. Int. J. Climatol..

[24] Tischner Z (2022). Survival and growth of microscopic fungi derived from tropical regions under future heat waves in the Pannonian Biogeographical Region. Fungal Biol..

[25] ElevationMap. (2020) Ethiopia, Uganda and Tanzania. *ElevationMap*. [online] Available from: https://elevationmap.net/. [Accessed 8 Jan 2021].

[26] Wickham H, Hester J, Francois R (2017). Readr: Read rectangular text data. R Package Version..

[27] Hijmans RJ, Phillips S, Leathwick J, Elith J, Hijmans MRJ (2017). Package ‘dismo’. Circles..

[28] O’Donnell MS, Ignizio DA (2012). Bioclimatic predictors for supporting ecological applications in the conterminous United States. US Geol. Surv. Data Ser..

[29] Mark, W. (2019). *MOHC HadGEM3-GC31-LL model output prepared for CMIP6 CFMIP. Version 20200403.* Earth System Grid Federation. 10.22033/ESGF/CMIP6.435

[30] Hijmans, R. J., et al. (2015) Package ‘raster’. *R Package.* 734. https://cran.r-project.org/web/packages/raster/index.html.

[31] Velásquez AC, Castroverde CDM, He SY (2018). Plant-pathogen warfare under changing climate conditions. Curr. Biol..

[32] Padfield D, Osullivan H, Pawar S (2021). rTPC and nlsmultstart: A new pipeline to fit thermal performance curves in r. Methods Ecol. Evol..

[33] Robinson, D. (2014) broom: An R package for converting statistical analysis objects into tidy data frames. *arXiv Preprint *arXiv:1412.3565*.* https://cran.r-project.org/web/packages/broom/index.html.

[34] Schoolfield RM, Sharpe P, Magnuson CE (1981). Non-linear regression of biological temperature-dependent rate models based on absolute reaction-rate theory. J. Theor. Biol..

[35] Halder, J. B. (2018) *Environmental influences on Fusarium Head Blight.* PhD thesis. Imperial College London. https://spiral.imperial.ac.uk/bitstream/10044/1/59001/1/Halder-JB-2018-PhD-Thesis.pdf.

[36] Boyce Thompson Institute (BTI). (2015) *Algae to Energy - Using and Re-using a Hemocytometer to Count Algae Cells.* BTI Curriculum Development Projects in Plant Biology Algae to Energy Hemocytometer Use. https://btiscience.org/wp-content/uploads/2015/12/e.-Algae-to-Energy-Counting-Algae-Cells.pdf.

[37] Bates, D. M. (2010) Lme4: Mixed-Effects Modeling with R. https://r-forge.r-project.org/scm/viewvc.php/*checkout*/www/lMMwR/lrgprt.pdf?revision=600&root=lme4&pathrev=601.

[38] Kuznetsova A, Brockhoff PB, Christensen RH (2017). lmerTest package: Tests in linear mixed effects models. J. Stat. Softw..

[39] Russell, L. (2018) emmeans: Estimated Marginal Means, Aka Least-Squares Means. R Package Version 1.3.0. https://cran.r-project.org/web/packages/emmeans/index.html.

[40] International Coffee Organisation (ICO). (2022) *Historical Data on the Global Coffee Trade.* https://www.ico.org/new_historical.asp.

[41] Waller, J. M., Bigger, M. & Hillocks, R. J. (2007) World coffee production. In: Anonymous Coffee pests, diseases and their management. [e-book], CABI Wallingford UK. pp. 17–33. 10.1079/9781845931292.0017.

[42] Dong F (2016). Effect of environmental factors on Fusarium population and associated trichothecenes in wheat grain grown in Jiangsu province, China. Int. J. Food Microbiol..

[43] Niederer M, Pankow W, Wiemken A (1992). Seasonal changes of soluble carbohydrates in mycorrhizas of Norway spruce and changes induced by exposure to frost and desiccation. Eur. J. For. Pathol..

[44] Cooke, R. C. & Whipps, J. M. (1993) *Ecophysiology of fungi*. [e-book], Blackwell Scientific Publications. https://www.wiley.com/en-us/Ecophysiology+of+Fungi-p-9780632021680.

[45] Weinstein RN, Montiel PO, Johnstone K (2000). Influence of growth temperature on lipid and soluble carbohydrate synthesis by fungi isolated from fellfield soil in the maritime Antarctic. Mycologia.

[46] Robinson CH (2001). Cold adaptation in Arctic and Antarctic fungi. New Phytol..

[47] Peck, L. D. (2023), *Seventy years of Fusarium wilt - coffee interactions: historical genomics reveals pathogen emergence and divergence*. [PhD thesis] Imperial College London.

[48] Lembrechts JJ (2020). SoilTemp: A global database of near-surface temperature. Glob Change Biol..

[49] Belachew K, Teferi D, Hundessa N, Tesfaye S (2016). The statue and management of Coffee wilt disease (*Gibberella xylarioides*) in Ethiopian Coffee production. J. Nat. Sci. Res..

[50] Tshilenge-Djim P, Kalonji-Mbuyi A, Tshilenge-Lukanda L (2011). Variability of pathogenicity in *Fusarium xylarioides* Steyaert: The causal agent of coffee wilt disease. J. Exper. Agric. Int..

