# Supplementary material for: Temperature contributes to host specialization of coffee wilt disease (Fusarium xylarioides) on arabica and robusta coffee crops
Source: Sci Rep. 2023 Jun 8;13:9327. doi: 10.1038/s41598-023-36474-w (PMC10250448; doi:10.1038/s41598-023-36474-w)
Supplement: Supplementary file 1 — Supplementary Information. [file 41598_2023_36474_MOESM1_ESM.docx]

**Zhang et al. Supplementary information**

**Table S1.** Information for arabica and robusta isolates of *F. xylarioides* used in growth assays. Stock of isolates was provided by CABI IMI culture collection.

| **Host** | **IMI number** | **Location of originality** | **Mean temperature (°C)** | **Mean precipitation (mm)** | **Year of isolation** |
| --- | --- | --- | --- | --- | --- |
| **Arabica** | 389563 | Karo Mariam, Ethiopia | 20.0 | 1815 | 2002 |
|  | 393593 | Illubabor, Ethiopia | 19.9 | 1788 | 2005 |
| **Robusta** | 392263 | Karimundi, Uganda | 22.8 | 1269 | 2000 |
|  | 392278 | Karagwe, Tanzania | 19.7 | 1062 | 2003 |

| **Fixed effects** | | | | | | | |
| --- | --- | --- | --- | --- | --- | --- | --- |
| **Form** | **Temperature (°C)** | | **Estimate** | **Standard error** | | **Lower CI** | **Upper CI** |
| **Arabica** | 10 | | 0.435 | 0.223 | | -0.027 | 0.897 |
|  | 15 | | 0.379 | 0.280 | | 0.003 | 0.755 |
|  | 20 | | 1.878 | 0.275 | | 1.515 | 2.242 |
|  | 25 | | 2.026 | 0.280 | | 1.650 | 2.402 |
|  | 30 | | 1.124 | 0.271 | | 0.771 | 1.476 |
|  | 35 | | 1.591 | 0.313 | | 1.129 | 2.053 |
|  | 40 | | 0.246 | 0.350 | | -0.309 | 0.800 |
| **Robusta** | 10 | | 0.295 | 0.325 | | -0.167 | 0.757 |
|  | 15 | | 0.272 | 0.396 | | -0.104 | 0.648 |
|  | 20 | | 2.976 | 0.389 | | 2.612 | 3.339 |
|  | 25 | | 3.373 | 0.396 | | 2.998 | 3.749 |
|  | 30 | | 1.887 | 0.383 | | 1.534 | 2.240 |
|  | 35 | | 2.047 | 0.442 | | 1.584 | 2.509 |
|  | 40 | | 0.615 | 0.495 | | 0.061 | 1.169 |
| **Random effects** | | | | | | | |
| **Groups** | | **Variance** | | | **Standard deviation** | | |
| **Strain** | | 7.759*10^-3^ | | | 0.0881 | | |
| **Round** | | 3.763*10^-8^ | | | 0.0002 | | |
| **Residual** | | 0.2935 | | | 0.5418 | | |

**Table S2.** Outputs for linear mixed-effect model for the mean mycelium growth rates of *F. xylarioides* (mm/day). The lower and upper CI represent confidence intervals for model predictions.

**Table S3.** Outputs for linear mixed-effect model for the sporulation rates of *F. xylarioides* (1000/mm^2^). The lower and upper CI represent confidence intervals for model predictions.

| **Fixed effects** | | | | | | | |
| --- | --- | --- | --- | --- | --- | --- | --- |
| **Form** | **Temperature (°C)** | | **Estimate** | **Standard error** | | **Lower CI** | **Upper CI** |
| **Arabica** | 10 | | 17.86 | 4.75 | | -5.05 | 40.76 |
|  | 15 | | 20.03 | 4.59 | | -6.16 | 46.23 |
|  | 20 | | 10.47 | 4.59 | | -15.73 | 36.68 |
|  | 25 | | 10.48 | 4.59 | | -15.72 | 36.69 |
|  | 30 | | 8.49 | 4.59 | | -17.71 | 34.69 |
|  | 35 | | -2.73 | 4.75 | | -25.63 | 20.18 |
|  | 40 | | 5.20 | 4.75 | | -17.70 | 28.11 |
| **Robusta** | 10 | | 4.17 | 4.75 | | -18.73 | 27.10 |
|  | 15 | | 5.76 | 4.59 | | -20.43 | 31.96 |
|  | 20 | | 4.80 | 4.59 | | -21.40 | 31.00 |
|  | 25 | | 8.01 | 4.59 | | -18.19 | 34.21 |
|  | 30 | | 5.69 | 4.59 | | -20.51 | 31.88 |
|  | 35 | | 6.43 | 4.75 | | -16.47 | 29.33 |
|  | 40 | | 5.80 | 4.75 | | -17.11 | 28.70 |
| **Random effects** | | | | | | | |
| **Groups** | | **Variance** | | | **Standard deviation** | | |
| **Strain** | | 6.36 | | | 2.52 | | |
| **Round** | | 33.05 | | | 5.75 | | |
| **Residual** | | 10.86 | | | 3.30 | | |

**Table S4.** Outputs for linear mixed-effect model for the spore gemination rates of *F. xylarioides*. The lower and upper CI represent confidence intervals for model predictions.

| **Fixed effects** | | | | | | | |
| --- | --- | --- | --- | --- | --- | --- | --- |
| **Form** | **Temperature (°C)** | | **Estimate** | **Standard error** | | **Lower CI** | **Upper CI** |
| **Arabica** | 10 | | 0.781 | 0.14412 | | 0.239 | 1.323 |
|  | 15 | | 0.758 | 0.08791 | | 0.097 | 1.420 |
|  | 20 | | 0.863 | 0.08791 | | 0.201 | 1.524 |
|  | 25 | | 0.857 | 0.08791 | | 0.196 | 1.518 |
|  | 30 | | 0.551 | 0.08791 | | -0.111 | 1.212 |
|  | 35 | | 0.696 | 0.09950 | | 0.154 | 1.238 |
|  | 40 | | 0.137 | 0.10540 | | -0.405 | 0.679 |
| **Robusta** | 10 | | 0.315 | 0.12124 | | -0.226 | 0.857 |
|  | 15 | | 0.497 | 0.12186 | | -0.167 | 1.158 |
|  | 20 | | 0.815 | 0.12186 | | 0.152 | 1.477 |
|  | 25 | | 0.794 | 0.12186 | | 0.132 | 1.455 |
|  | 30 | | 0.571 | 0.12186 | | -0.091 | 1.232 |
|  | 35 | | 0.662 | 0.14071 | | 0.120 | 1.204 |
|  | 40 | | 0.182 | 0.14071 | | -0.360 | 0.724 |
| **Random effects** | | | | | | | |
| **Groups** | | **Variance** | | | **Standard deviation** | | |
| **Strain** | | 0.00480 | | | 0.06927 | | |
| **Round** | | 0.02624 | | | 0.16197 | | |
| **Residual** | | 0.02970 | | | 0.17234 | | |


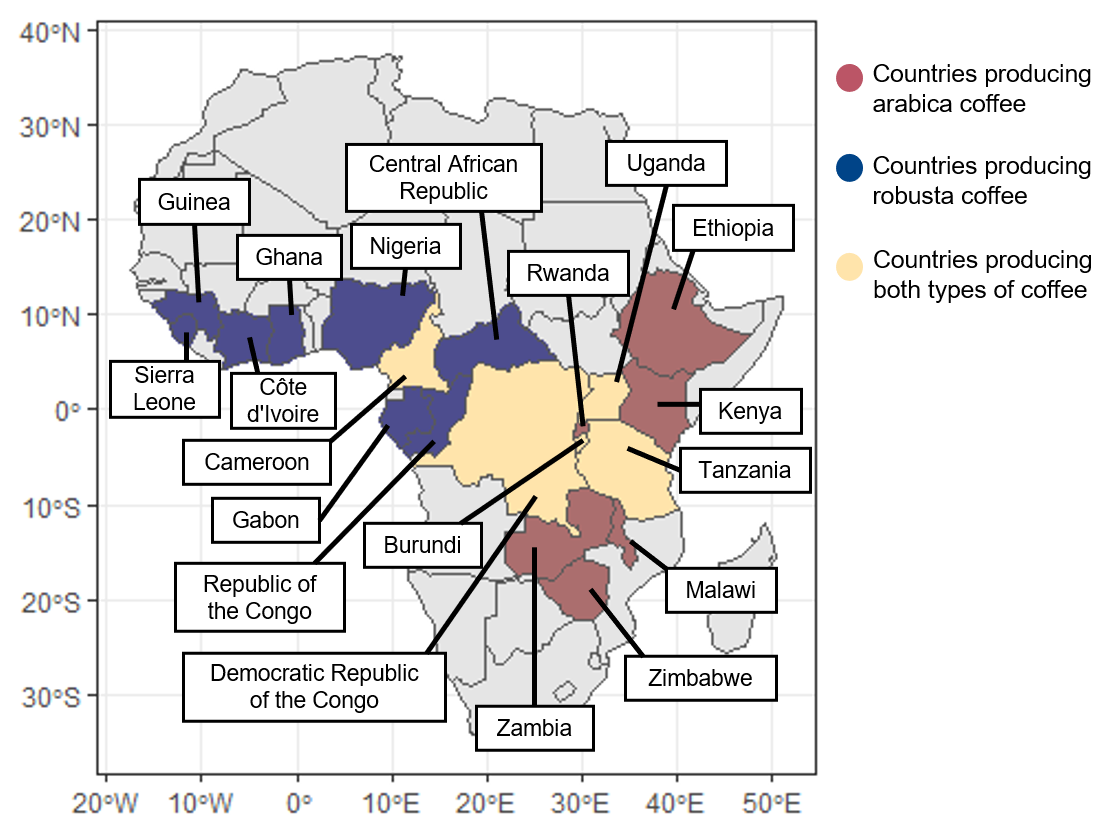


**Figure S1.** Distribution of major coffee-producing countries in Africa, the colour of countries represent the type of coffee they produce.

**Supplementary information S1. Climatic modelling incorporating precipitation patterns**

*Statistical modelling*. We extracted the bioclimatic variables of villages with incidences of the disease in 2002, using their latitude and longitude data in Oduor et al (2003), with R package ‘raster’ (Hijmans et al, 2015). To assess the effect of mean and extreme temperature and precipitation on severity of CWD, we fitted the severity records of these villages as response variables into binomial generalised linear-regression models (GLM). The original predictors for the models included bio1 (mean monthly temperature), bio5 (maximum monthly temperature), bio6 (minimum monthly temperature), bio12 (annual precipitation), bio13 (maximum monthly precipitation), and bio14 (minimum monthly precipitation); but bio5 and bio6 were shown to be significantly correlated with bio1 (Pearson’s product-moment correlation: bio-bio5: t_30,316_=302.04, p<0.001, correlation coefficient=0.866; bio1-bio6: t_30,316_=148.52, p<0.001, Correlation coefficient=0.649), and bio13 was correlated with bio12 (t_30,316_=235.74, p<0.001, correlation coefficient=0.804). Thus, the final model only incorporated bio1, bio12 and bio14 as predictors to avoid intercorrelation. The models were assessed for their coefficient of determination (adj R^2^) and effect of each predictor on severity of the host-specific forms, and then used to predict the potential severity of arabica and robusta forms across all African coffee-producing countries using past (1970-2000) WorldClim data. We also predicted potential future severity under project climate changes for the region using future CMIP6 (2060-2080) bioclimatic data by predicting values for the model parameters, but fed with future temperatures instead of past ones, and compared the change between past and future predictions.

*Model results*. Our binomial GLM explained little of the variation in severity of the arabica form (adj R_2_ = -0.032), and none of the predictor variables were significantly correlated with severity of the arabica form (bio1: χ^2^_1_=0.179, Est=-0.141, p=0.671; bio12: χ^2^_1_=0.005, Est=-0.000189, p=0.946; bio14: χ^2^_1_=0.506, Est=0.050, p=0.477). The GLM model explained approximately a sixth of the variation in severity of the robusta form (adj R_2_ = 0.159). Bio14 had a significant positive correlation with severity of this form (χ^2^_1_=7.053, Est=0.076, p=0.018), while bio1 and bio12 were not significantly correlated with the response variable (bio1: χ^2^_1_=0.100, Est=-0.174, p=0.752; bio12: χ^2^_1_=2.42, Est=-0.00273, p=0.120). Despite different levels of significance, both models predicted bio1 and bio12 to be negatively correlated, while bio14 was positively correlated with severity of the corresponding host-specific form.

*Past severity*. WorldClim (1970-2000) data were used to extrapolate the models reported above to predict past spatial variation in severity across coffee-producing regions of Africa (between 20°N and 20°S, Figure S1). Predicted past severity of the arabica form was generally low within the sampled region (mean=1.91%, SD=5.97%), but some regions were predicted to have high severity (>20%). The major arabica-coffee-producing regions predicted to have high severity include Ethiopian Mountains, southwest Kenya, southeast Uganda, and most pronouncedly, central Democratic Republic of the Congo (DRC), where severity up to 80% was predicted (ICO, 2022) (Figure S2a). Though not major producers of arabica coffee by ICO (2022), the southern coast of Liberia and eastern coast of Madagascar were also predicted to have severity >20% (Figure S2a). In contrast, the robusta form was predicted to have higher average severity than the arabica form (mean=20.79%, SD=18.40%). Like the other model, some regions were also predicted to have high severity (>50%), especially southern Uganda and northern DRC, which are major producers of robusta coffee (ICO, 2022), and its severity can reach up to 100% in these regions (Figure S2b). Despite not major robusta-coffee producers (ICO, 2022), southwestern Namibia and Kenya, Ethiopian Mountains, and southern Liberia were also predicted to have high severity for the robusta form over 50% (Figure S2b).


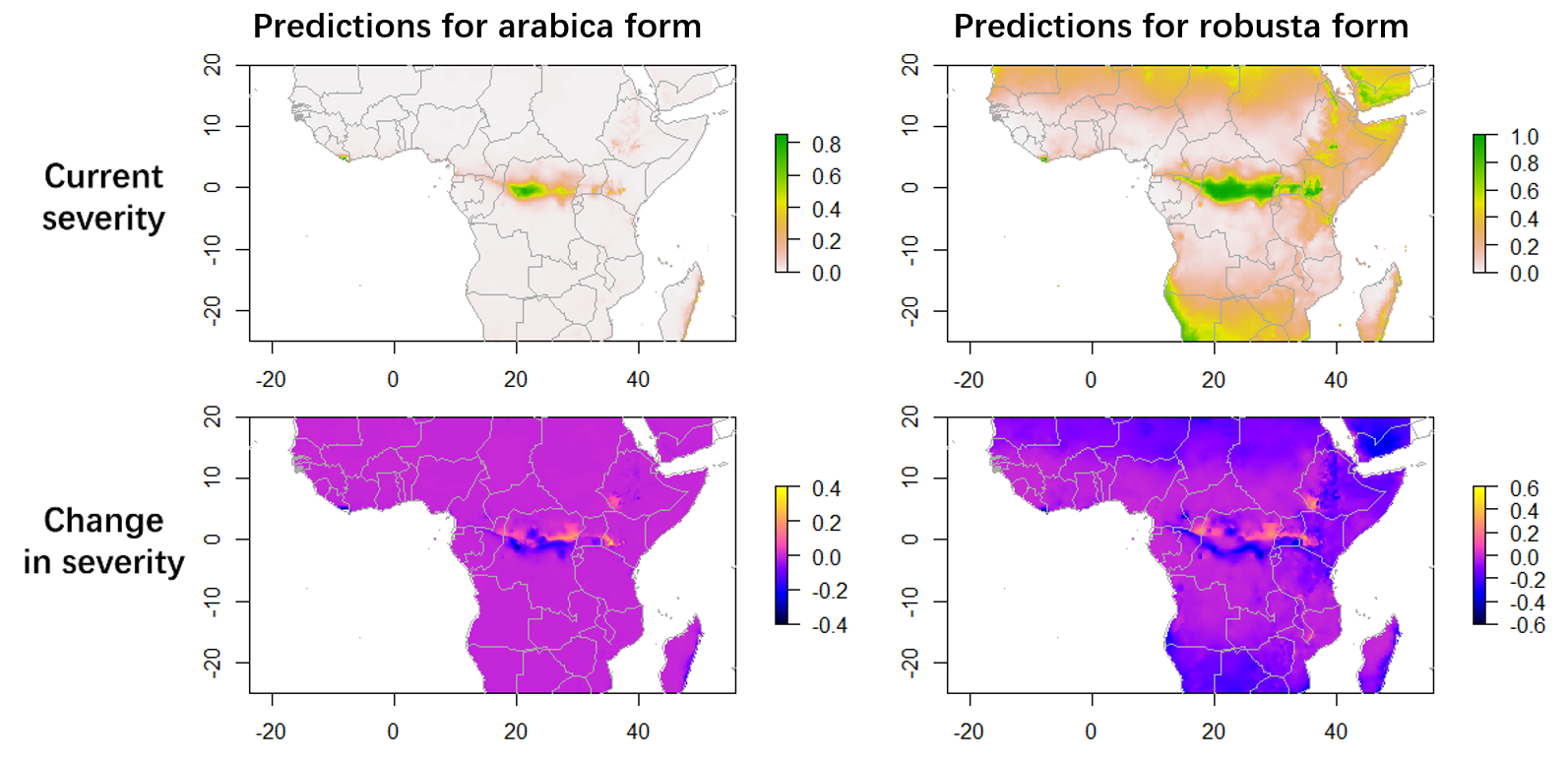


**Figure.S2.** Predicted severity for arabica and robusta forms of *F. xylarioides* in major coffee-growing countries of Africa, using binomial models incorporating bio1 (mean monthly temperature), bio12 (annual precipitation) and bio14 (minimum monthly precipitation) as predictors. **(a)** Predicted severity for arabica form under past climatic data (1970-2000) (mean = 1.91%). **(b)** Predicted severity for robusta form under past climatic data (mean = 20.79%). **(c)** Change in predicted severity for arabica form between past and future (2060-2080) climatic data (mean change = -0.47%). **(d)** Change in predicted severity for robusta form between past and future climatic data (mean change = -7.69%).

*Future severity*. CMIP6 (2060-2080) bioclimatic data were used to extrapolate the models to predict future potential severity across coffee-producing regions of Africa based on future temperature projections. The severity of the arabica form was predicted to decrease by approximately a quarter from past (1970-2000) to future (2060-2080) climate (mean change = -0.47%, Welch Two Sample t_130,803_=-15.36, p<0.001, Figure S2c). Despite the overall decrease in this area, especially in equatorial Congo Basin, predicted to experience reduction over 20%, southwestern Ethiopia, southwestern Kenya and northern DRC and Republic of the Congo were estimated to have an increase in severity (>20%) compared to past predictions (Figure S2c), and these countries are all important producers of arabica coffee (ICO, 2022; Figure S1). Similarly, the robusta form was also predicted to decrease in severity for future predictions (mean change = -7.69%, Welch Two Sample t_127,304_=-84.08, p<0.001), especially around equatorial DRC, northern Republic of the Congo and southern Uganda (Figure S2d). However, some major robusta-coffee-producing regions were predicted to have increased severity (>20%), including northern DRC and Republic of the Congo and central Uganda (Figure S2d). Though not producers of robusta coffee (Waller et al, 2007; ICO, 2022), the southwestern parts of Kenya and Ethiopia were also projected to experience increased severity of this form (Figure S2d).
